# Characterization of Family IV UDG from *Aeropyrum pernix* and Its Application in Hot-Start PCR by Family B DNA Polymerase

**DOI:** 10.1371/journal.pone.0027248

**Published:** 2011-11-08

**Authors:** Xi-Peng Liu, Jian-Hua Liu

**Affiliations:** State Key Laboratory of Microbial Metabolism, School of Life Sciences & Biotechnology, Shanghai Jiao Tong University, Shanghai, China; New England Biolabs, Inc., United States of America

## Abstract

Recombinant uracil-DNA glycosylase (UDG) from *Aeropyrum pernix* (*A. pernix*) was expressed in *E. coli*. The biochemical characteristics of *A. pernix* UDG (ApeUDG) were studied using oligonucleotides carrying a deoxyuracil (dU) base. The optimal temperature range and pH value for dU removal by ApeUDG were 55–65°C and pH 9.0, respectively. The removal of dU was inhibited by the divalent ions of Zn, Cu, Co, Ni, and Mn, as well as a high concentration of NaCl. The opposite base in the complementary strand affected the dU removal by ApeUDG as follows: U/C≈U/G>U/T≈U/AP≈U/->U/U≈U/I>U/A. The phosphorothioate around dU strongly inhibited dU removal by ApeUDG. Based on the above biochemical characteristics and the conservation of amino acid residues, ApeUDG was determined to belong to the IV UDG family. ApeUDG increased the yield of PCR by Pfu DNA polymerase via the removal of dU in amplified DNA. Using the dU-carrying oligonucleotide as an inhibitor and ApeUDG as an activator of Pfu DNA polymerase, the yield of undesired DNA fragments, such as primer-dimer, was significantly decreased, and the yield of the PCR target fragment was increased. This strategy, which aims to amplify the target gene with high specificity and yield, can be applied to all family B DNA polymerases.

## Introduction

Deamination of cytosine in DNA leads to dU/dG damage and deamination of dCTP results in dUTP that can be misincorporated into the genome during replication in the form of dUMP. The high temperature rate constants for the spontaneous deamination of cytosine in DNA and dCTP are several orders of magnitude higher than those at more moderate temperatures [1]. Hyperthermophiles live at temperatures above 80°C, so hyperthermophilic microbes face a serious high-temperature threat, and consequently develop several strategies for confronting dU damage.

First, dUTPase can hydrolyze harmful dUTP [2], [3]. Second, various UDGs remove dU from DNA, and other proteins complete the base excision repair [4]–[6]. Third, the family B DNA polymerase specifically binds to the mutant-base dU in the template-strand and stops ahead of dU damage, avoiding the incorporation of dAMP opposite to dU [7]–[9]. These proteins are thought to be the main participants in the processing of dU damage.

As an important DNA repair protein, UDG removes the dU residues in the genome. UDGs have recently been characterized and divided into six families according to their amino acid sequence and substrate specificity: I (UNG family, typified by *E. coli* UDG) [10], II (MUG/TDG family) [4], [11], III (SMUG family) [12], IV (thermostable UDG family) [5], V (PaUDG-b family) [6], and VI (HDG family) [13]. These UDGs have two conserved active-site motifs: motifs I and II [6]. Motif I is responsible for the activation of the catalytic water molecule. Motif II interacts with the minor groove once the base is flipped out into the active site and stabilizes the protein-DNA complex.

Generally, more than one dU removal property is possessed by an organism. Hyperthermophiles have at least one of the following UDG families: II, IV, and V. Previous research showed that the mutation rate of G:C to A:T in *Thermus thermophilus* is significantly increased in the following order: *udg*A and *udg*B double mutant >* udg*A mutant > *udg*B mutant [14], indicating that UDG families IV and V commonly function *in vivo* to remove dU in DNA.

Many DNA polymerases, including family B DNA polymerase, are used in the amplification of DNA by PCR. Some efforts have been made to improve this DNA amplification technology in terms of yield, specificity, and length of the amplified DNA. During PCR, the high temperature leads to the generation of dUTP and dU through the deamination of dCTP and dC base, which is harmful for PCR by family B DNA polymerase and results in less product yield, and even failure of amplification.

dUTPase can increase the yield and length of the product in PCR by family B DNA polymerase via deletion of harmful dUTP [2]. Another problem is that undesired DNA synthesis can sometimes occur during the PCR set-up. There are two types of undesired DNA synthesis: mispriming on less than specific sites in the template and the formation of a primer dimer. Some methods have been developed to prevent unwanted DNA synthesis during PCR. The strategy is based on the blocking of DNA synthesis at room temperature by physical separation of PCR components into two parts, antibody to polymerase, chemical modification of polymerase and reaction buffer, usage of special primers, or cold sensitive mutant of polymerase [15]–[23].

*A. pernix* is a strictly aerobic archaea that inhabits environments ranging from 90 to 95°C [24]. In order to elucidate the pathway of dU excision repair in *A. pernix*, we cloned two genes potentially involved in the repair of dU: thymine-DNA glycosylase (ORF ape_0875.1) and uracil-DNA glycosylase (ORF ape_0427.1). We have characterized the enzymic activity of thymine-DNA glycosylase [25]. Amino acid alignment showed that ApeUDG has a high degree of similarity to family IV UDG and a low degree of similarity to family V UDG. Our results confirmed that ApeUDG can efficiently remove dU residues from both single- and double-stranded DNA.

ApeUDG can promote PCR by family B DNA polymerase via removal of dU from DNA and release of the tightly bound DNA polymerase from dU. In combination with dU-carrying oligonucleotide, a hot-start PCR is developed using ApeUDG and Pfu DNA polymerase. The strategy blocks the family B DNA polymerase at room temperature using a dU-carrying oligonucleotide, after which the block is deleted through the removal of dU by ApeUDG so that PCR starts.

## Materials and Methods

### Materials

Restriction endonucleases, *Pfu* DNA polymerase, and T4 DNA ligase were purchased from Fermentas. Expression vectors, *E. coli* strain BL21 (DE3), and Ni-NTA His•Bind® Resin were purchased from Novagen. Oligonucleotides were synthesized by TaKaRa (Dalian, China). *A. pernix* K5 strain was obtained from Japan Collection of Microorganisms (JCM, Japan). All other chemicals and reagents were of analytical grade.

### Expression and purification of ApeUDG

The *apeudg* gene (ape_0427.1) was amplified from *A. pernix* genomic DNA by PCR using forward primer (5′CCCCC***CATAtg***gctggcagcaggctcag3′) and reverse primer (5′CCCCC***GAATTc***taggagtctacatcaccgc3′). The amplified DNA fragments were inserted into pET28a vector by T4 DNA ligase, generating expression plasmid pET28a-*apeudg*.

Expression and purification of recombinant protein ApeUDG were performed as described [25]. *E. coli* BL21 (DE3) harboring pET28a-*apeudg* was induced with IPTG to express the recombinant ApeUDG. Induced-bacteria were lysed by sonication. The lysate was incubated at 65°C for 30 min before clarification by centrifugation. The clarified lysate was used to purify recombinant ApeUDG through Ni-NTA His•Bind® Resin column. All eluates were fractionally collected and analyzed by 15% SDS-PAGE. The purified ApeUDG was stored in small aliquots at −20°C.

### Characterization of the DNA glycosylase of ApeUDG

Oligonucleotide of 5′FAM-dU (5′FAM-GCTGCAGGAA **dU** TCGATATCAA3′) labeled with the fluorescent group of FAM at the 5′ end was used as a substrate in the glycosylase assay. The ds substrates were prepared by annealing 5′FAM-dU with the unlabeled complementary strands at a mole ratio of 1∶1.5. To characterize the effect of phosphorothioate on the excision of dU by ApeUDG, five successive phosphorothioates near dU (2 upstream and 2 downstream of dU) were introduced.

All reactions (20 µL) were incubated at 50°C for 15 min unless specified. After incubation, the reaction was treated with 0.1 M NaOH at 90°C for 10 min and neutralized with HCl. Reaction products were resolved by 20% 8 M urea denatured PAGE after the addition of an equal volume of loading buffer (95% formamide, 50 mM EDTA, 0.02% bromophenol blue, and 0.02% xylene cyanol), and then visualized by Phosphorimager (FL5100, Fujii).

Prior to optimization, the standard reaction buffer contained 20 mM Tris-HCl pH 8.0, 50 mM NaCl, 1 mM EDTA, 1 mM DTT, and 100 ng/µL BSA. To optimize pH values, the assays were performed in buffers (20 mM) of varying pH: imidazole-HCl with pH 5.0, 5.5, 6.0, or 6.5, Tris-HCl with pH 7.0, 7.5, 8.0, or 8.5, and glycine/NaOH with pH 9.0, 9.5, 10.0, or 10.5. Following pH optimization, all of the subsequent reactions were performed in 20 mM glycine-NaOH, with pH 9.0.

An experiment to determine the effect of ionic strength on glycosylase was performed in buffers containing 0.05, 0.1, 0.2, 0.3, 0.4, 0.5, 0.7, and 1 M NaCl. An experiment to determine the effect of divalent ions on glycosylase was performed in buffers containing 5 mM of different divalent ions. Following optimization, all reactions were performed in an optimal assay buffer containing 20 mM glycine-NaOH pH 9.0, 50 mM NaCl, 1 mM EDTA, 1 mM DTT, and 100 ng/µL BSA.

### Enhancement of PCR by family B DNA polymerase

The ORF of ape_0119 from *A. pernix* was amplified by several family B DNA polymerases to characterize the improvement of PCR by ApeUDG and dU-carrying oligonucleotide. The dU-carrying oligo (dU-oligo, 5′TdUTTTGGAATGCCTGCAG GAATTCGATATCAGCATTCC3′) forms a stem-loop structure with a five-nucleotide length 5′ tail. PCR reaction (50 µL) containing 5 ng of *A. pernix* genomic DNA was subjected to predenaturation for 5 min at 95°C and 30 cycles at 94°C for 30 s, 55°C for 30 s, and 72°C for 90 s, followed by a 5-min extension at 72°C. The linear pDEST17 vector (about 4.6 kb) was amplified to check the improvement of amplification of the long DNA fragment by ApeUDG and dU-oligo. PCR reaction (50 µL) was carried out using 1 ng of circular pDEST17 DNA at the following conditions: 5 min predenaturation at 95°C, 20 cycles at 94°C for 30 s, 55°C for 30 s, and 72°C for 6 min, and a final extension at 72°C for 10 min. To block polymerase activity, dU-oligo was premixed with DNA polymerase in PCR buffers and placed at room temperature for 15 min before the start of PCR by the addition of ApeUDG.

## Results

### Expression and purification of ApeUDG

**Figure 1:**
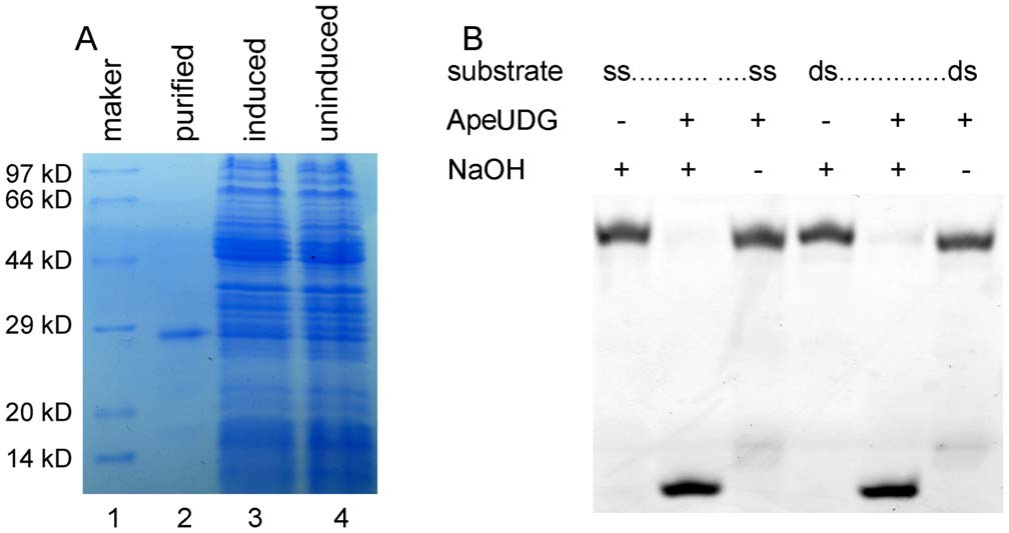
Removal activity of dU associated with ApeUDG. (A) 15% SDS-PAGE analysis of recombinant ApeUDG recovered from *E. coli* extracts. The gel was stained with Coomassie blue R-250. Lane 1: molecular weight marker; lane 2: purified recombinant protein; lane 3: induced *E. coli* total proteins; and lane 4: uninduced *E. coli* total proteins. (B) Removal of dU from ss and ds DNA by ApeUDG. Approximately 10 ng ApeUDG was incubated with 1 µM ss or ds DNA (G/U) for 15 min at 50°C.

After purification by native Ni-NTA His•Bind® Resin column chromatography, ApeUDG becomes electrophoretically homogenous, as demonstrated by 15% SDS-PAGE (Fig. 1A). Purified ApeUDG has a strong DNA glycosylase activity that removes dU from ss and ds DNA, resulting in the generation of an apurinic/apyrimidinic (AP) site, which can be cleaved by treatment with hot-alkali (Fig. 1B). However, no distinct AP lyase activity is detected for ApeUDG (Fig. 1B).

### The optimal reaction of dU removal by ApeUDG

**Figure 2:**
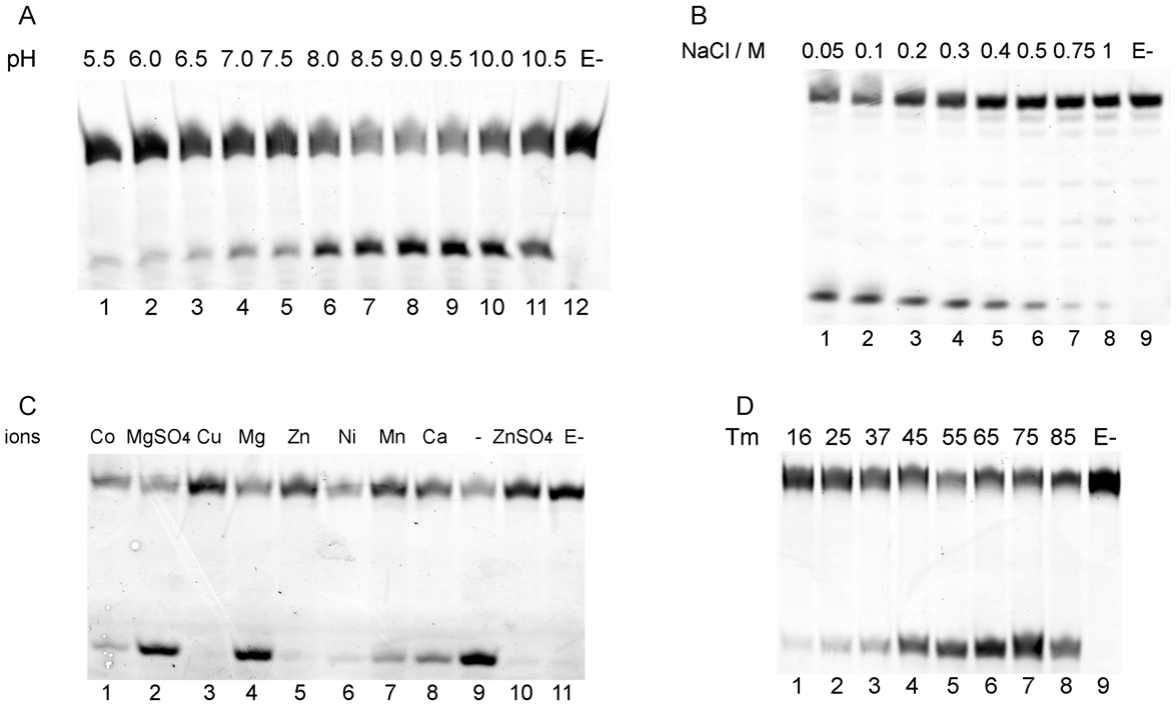
Effect of (A) pH value, (B) NaCl concentration, (C) divalent ions, and (D) reaction temperature on dU removal by ApeUDG. About 1 ng ApeUDG and 1 µM dU-carrying ss DNA were incubated at 50°C for 15 min in assay buffer with various pH value, ion strength, or divalent ions. 1 ng ApeUDG and 1 µM dU-carrying ss DNA were incubated at various temperatures for 15 min in an optimal assay buffer.

The glycosylase activity of ApeUDG was characterized in the reaction buffers with pH values ranging from 5.5 to 10.5. The ApeUDG has high dU cleavage activity at pH values ranging from 8.0 to 10.5, with the highest removal of dU from ss DNA at pH 9.0 (Fig. 2A).

A high concentration of NaCl inhibits the glycosylase of ApeUDG to some extent (Fig. 2B). When the concentration of NaCl in the reaction buffer is higher than 300 mM, the glycosylase activity is decreased by more than 50%.

Divalent ions have different effects on the removal of dU from DNA by ApeUDG (Fig. 2C). Mg^2+^ has no distinct effect on the activity of ApeUDG, whereas Ca^2+^, Co^2+^, and Mn^2+^ show some inhibition of the reaction. Ni^2+^, Zn^2+^, and Cu^2+^ almost completely inhibit the enzymic activity. Meanwhile, the anions have no distinct effect on the removal of dU (Fig. 2C, lanes 2 and 10).

The reaction temperature optimization shows that ApeUDG has higher activity at high temperatures ranging from 45 to 85°C and less activity at low temperatures ranging from 16 to 37°C (Fig. 2D). This result is consistent with the growth temperature of *A. pernix*.

### The substrate specificity of ApeUDG

**Figure 3:**
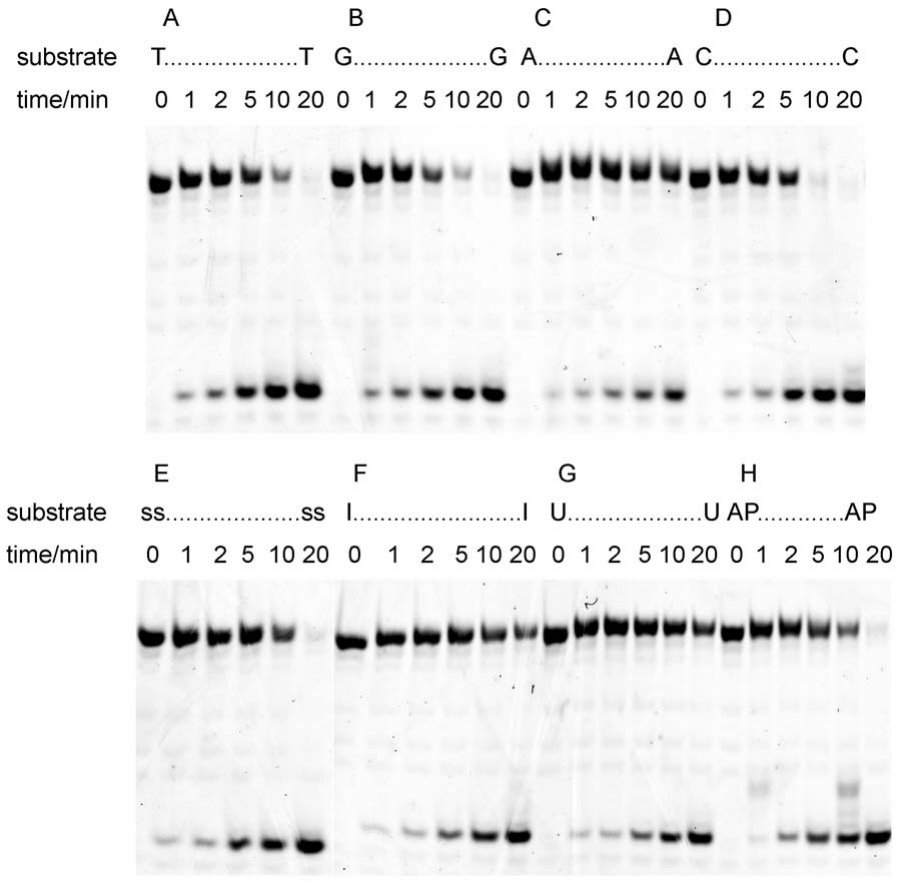
The excision specificity of ApeUDG on ds DNA substrate. Approximately 5 ng recombinant ApeUDG was incubated with 1 µM ss or ds DNA with various bases opposite dU at 50°C for different times in an optimal assay buffer.

Our results demonstrate that the recombinant ApeUDG can efficiently remove dU from both ss and ds oligonucleotides (Fig. 3). The bases (A, T, C, G, U, I, and AP site) opposite dU have some effect on the removal of dU in ds oligonucleotides by ApeUDG. The removal of dU opposite C and G is the most efficient (Figs. 3B and 3D). The removal of dU opposite T and AP site is comparable, with similar excision efficiency to ss DNA (Figs. 3A, 3E, and 3H). The removal of dU is least efficient if the opposite bases are A, I, and U (Figs. 3C, 3F, and 3G). Of course, maybe the uracil base opposite U is removed and the U/U pair is converted to the U/AP pair.

### The inhibition of phosphorothioates on dU removal

**Figure 4:**
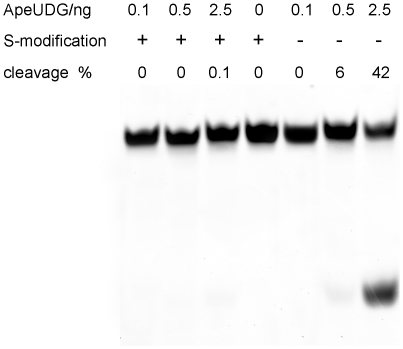
Inhibition of phosphorothioates near dU on the removal of dU by ApeUDG. 0.2 ng, 1 ng, and 5 ng of ApeUDG were incubated with 1 µM ds (dU/A) DNA with or without phosphorothiate modification at 50°C for 30 min.

Because family I UDG interacts with phosphates around dU [26], we changed some phosphates into phosphorothioates in the dU-carrying strand, and then characterized its effect on the removal of dU by ApeUDG. The introduction of five phosphorothiates around dU (from +3 to −2 phosphodiester groups of dU position) strongly decreases the dU removal by ApeUDG (Fig. 4), suggesting that the phosphorothiates hinder ApeUDG to flip out the dU base into the catalytic center for cleavage of the glycosyl bond.

### The effect of ApeUDG and dU-carrying oligonucleotide on PCR by Pfu DNA polymerase

**Figure 5:**
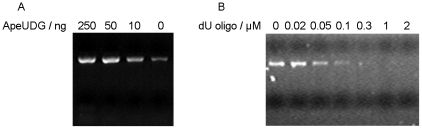
Effect of dU-carrying oligonucleotide and ApeUDG on PCR by Pfu DNA polymerase. The standard PCR of ape_0119 was performed in the presence of various amounts of (A) ApeUDG or (B) dU-carrying oligonucleotide. Amplified products were resolved in 1% agarose gel, along with a molecular weight standard DNA ladder (M).

Pfu DNA polymerase is more commonly used for PCR than Taq polymerase when high-fidelity amplification is required. During PCR reaction, deamination of dC and incorporation of dUMP results in the generation of uracil in DNA. Pfu DNA polymerase tightly binds dU in DNA and leads to the cessation of DNA extension in DNA synthesis [7]–[9], [27], [28]. We tested the effect of dU in DNA on the yield of fragments amplified by Pfu DNA polymerase using a dU-carrying oligonucleotide (dU-oligo). It has been previously demonstrated that Pfu DNA polymerase prefers to bind the dU nucleotide located on the ssDNA tail of ds DNA (dU with 4 bases distance to the adjacent dsDNA terminus) [9]. Therefore, a stem-loop oligonucleotide with a single-stranded 5′ tail where a dU nucleotide is positioned 4 bases from the stem's terminus is used as the dU-oligo to characterize its inhibition on PCR by Pfu DNA polymerase. The addition of dU-oligo strongly inhibits PCR by Pfu DNA polymerase (Fig. 5B). No distinct inhibition on PCR is observed when only 0.02 µM of dU-oligo is added under the same conditions. The inhibition becomes very clear when more than 0.02 µM dU-oligo is present during PCR. PCR is completely inhibited and does not synthesize any DNA fragment when more than 0.1 µM dU-oligo is added.

The dU in DNA causes Pfu DNA polymerase to stall due to polymerase's tight binding to dU and leads to less amplification yield. Considering that ApeUDG removes dU, the blocked Pfu DNA polymerase by dU in DNA could be relieved by ApeUDG. Our results shows that the use of thermostable ApeUDG largely improves the yield of PCR by Pfu DNA polymerase, suggesting that the activity of Pfu DNA polymerase is indeed blocked by dU in DNA generated during the PCR reaction, and that ApeUDG can efficiently remove dU from DNA (Fig. 5A).

### Effect of ApeUDG on the fidelity of amplification by Pfu DNA polymerase

Although the efficiency of amplification is increased by the use of ApeUDG (Figure 5A), the fidelity of DNA synthesis is probably decreased because of the removal of uracil in DNA. The activity of ApeUDG removes uracil, leaving AP-sites in the template strand that will be by-passed and incorporated into any one of four nucleotides by Pfu DNA polymerase. This is likely to decrease the fidelity of the synthesis. Therefore, the error rate of the reaction should be measured under these conditions.

The fidelity of PCR reaction actually decreases a little in the presence of ApeUDG, as confirmed by lacI-based PCR fidelity assay (Table 1) [29], indicating the 3′ to 5′ exonuclease proofreading activity does not perfectly function in correcting nucleotide misincorporations opposite an AP-site. Endonuclease IV (endoIV) can cleave the harmful AP-site, and Pfu DNA polymerase subsequently synthesizes DNA from the cleaved 3′ OH and generates a correct full-length strand. Therefore, it would restore, and even improve, the decreased fidelity due to the AP-site derived from the removal of uracil by UDG.

**Table 1:** Error rate of PCR by DNA polymerase in the presence of UDG and/or endo IV^a^.

Polymerase	Pfu DNA polymerase				Taq DNA polymerase			
Protein factor	-	UDG	endoIV	UDG+endoIV	-	UDG	endoIV	UDG+endoIV
Product/target	1853	5235	1902	5436	12845	12324	11673	12593
Doublings^b^	10.8	12.3	10.9	12.4	13.6	13.6	13.5	13.6
Mf^c^ (%±SD)	0.64±0.1	1.21±0.2	0.61±0.1	0.78+0.1	4.6±0.5	5.2±0.9	1.4±0.3	1.2±0.2
*ER* d (×10^−6^±SD)	1.7±0.3	2.8±0.5	1.6±0.3	1.8±0.2	9.6±1	10.8±2	3.0±0.6	2.6±0.4

a The fidelity of DNA replication during PCR supplied with UDG and/or EndoIV was measured using a previously described assay [29]. Briefly, a 1.9-kb sequence encoding *lacIOZ*α was PCR -amplified using primers containing 5′*Eco*RI restriction sites and a 1-ng *lacIOZ*α target. The PCR mixtures (50 µL) were subjected to predenaturation of 5 min at 95°C and 30 cycles at 94°C for 30 s, 55°C for 30 s, and 72°C for 3 min, followed by a 5-min extension at 72°C. The amplified fragments were digested with *Eco*RI, purified by gel electrophoresis, and ligated into λgt10 arms. The ligation reactions were packaged, and the λ phage was used to infect an α-complementing *Escherichia coli* host strain. Aliquots of infected cells were plated on duplicate LB plates with top agar containing either X-gal (1 mg/mL) or X-gal plus IPTG (1.5 mM).

b Template doublings (*d*) are determined using the equation 2*^d^* = (amount of PCR product)/(amount of starting target).

c Mutant frequencies (*mf*) are determined by dividing the total number of blue plaques (*lacI*– mutants) on the X-gal plates by the total number of plaques containing a functional *lacZ*α sequence (blue plaques on X-gal plus IPTG plates).

d Error rates are calculated using the equation *ER* = *mf*/(*bp·d*), where *mf* is the mutation frequency, *bp* is the number of detectable sites in *lacI* ( = 349), and *d* is the number of template doublings.

The lacI-based PCR fidelity assay confirms that the use of *A. pernix* endo IV and UDG increases the fidelity of PCR by Pfu DNA polymerase (Table 1). The following PCR reactions were performed in the presence of *A. pernix* endoIV (1 ng/µL). EndoIV and UDG from *A. pernix* also increase the fidelity of PCR by Taq DNA polymerases (Table 1), one family A DNA polymerase.

Although only endoIV is added into PCR mixtures, the fidelity of Taq DNA polymerase is largely increased. This is because endoIV possesses a 3′ exonuclease with a preference for mismatched bases besides the endonuclease activity on the AP site. The 3′ exonuclease of endoIV can supply the deficient proofreading activity of Taq DNA polymerase and improve the fidelity of DNA synthesis. Moreover, the combination of UDG and endoIV (AP endonuclease activity) can correct the error of the uracil base derived from the deamination of dC. Hence, the fidelity of Taq DNA polymerase can be increased by the combined use of thermostable UDG and endoIV.

### Hot-start PCR of different length fragments

**Figure 6:**
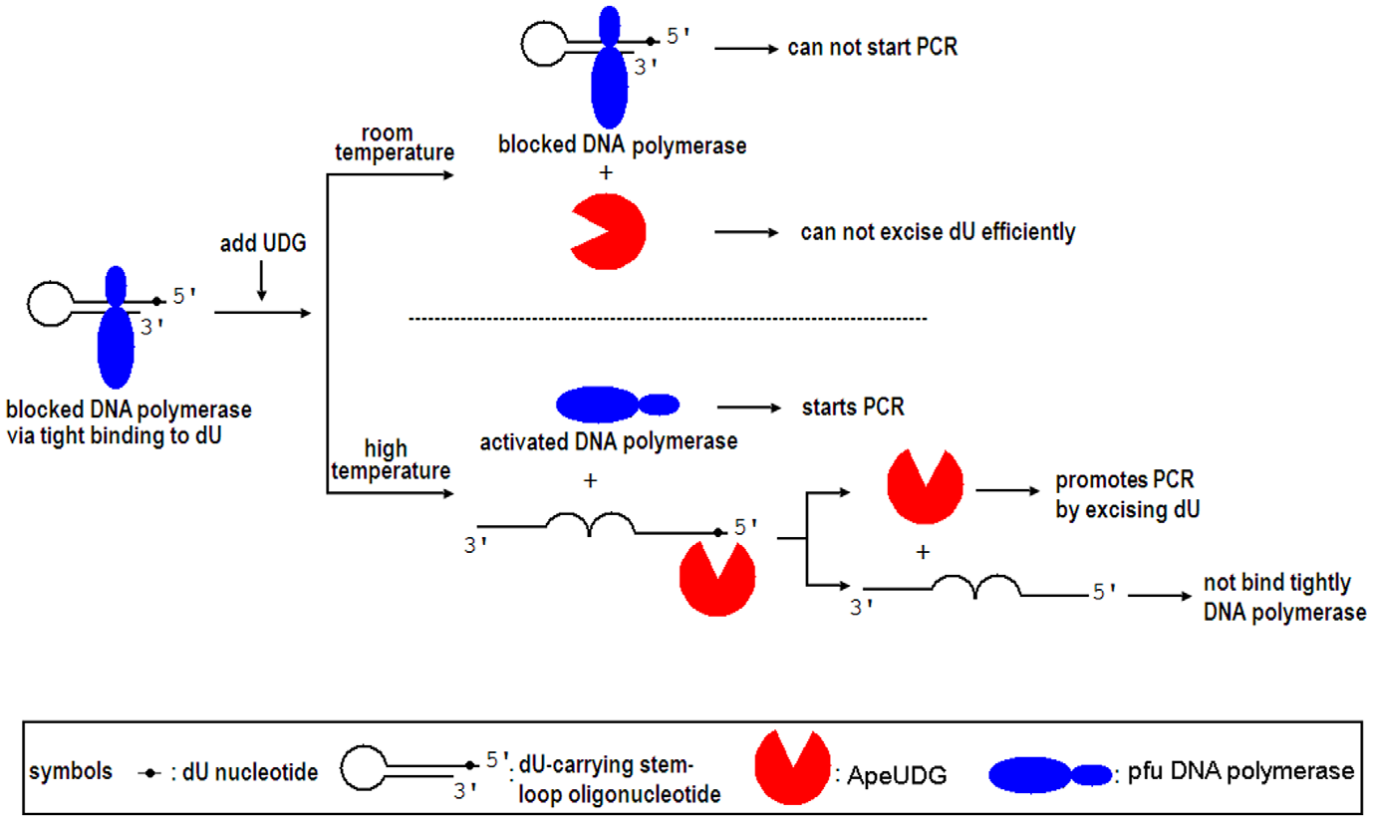
Schematic cartoon of hot-start PCR by Pfu DNA polymerase. The symbols for Pfu DNA polymerase, ApeUDG, dU-carrying stem-loop oligonucleotide, and dU nucleotide are listed at the bottom of figure.

The scheme involves a hot-start PCR to obtain the highest yield of target fragment and lowest generation of undesired fragments (Fig. 6). In the above experiment, the amplification yield can be improved by ApeUDG in PCR catalyzed by Pfu DNA polymerase (Fig. 5A). This function of ApeUDG provides a base for high yield amplification by family B DNA polymerase. The inclusion of *A. pernix* endoIV in the PCR reaction can eliminate possible base mutations due to the AP site being derived from the removal of dU by ApeUDG, thereby guaranteeing PCR fidelity.

However, another problem of PCR is that unspecific DNA fragment(s) and/or primer-dimers are sometimes generated. These unspecific fragments and primer-dimers are derived from misannealed primer-template/primer-primer, which are amplified by DNA polymerase at low temperatures. In order to avoid DNA synthesis at low temperatures, a short dU-carrying oligonucleotide is used to block family B DNA polymerase in our strategy. The blocked polymerase is activated by both ApeUDG and heating.

After incubation at high temperatures, the stem-loop of dU-carrying oligonucleotide is disrupted, and most of the polymerase is dissociated from the dU-carrying oligonucleotide. dU is then removed from the dU-carrying oligonucleotide by ApeUDG, making it unable to block the polymerase.

The completely activated polymerase goes on to catalyze DNA synthesis in the form of PCR. At low temperatures, such as room temperature, DNA polymerase cannot be efficiently activated, so the generation of primer-dimers and unspecific fragments is largely decreased. After the removal of dU from the dU-carrying oligonucleotide, ApeUDG can improve the amplification yield by durative removal of dU from amplified fragments during the entire PCR process. The cleaved dU-carrying oligonucleotide can be easily removed together with the gene-specific primers by the purification of the PCR product using a commercial column. Therefore, our hot-start PCR has no restrictions on downstream applications.

**Figure 7:**
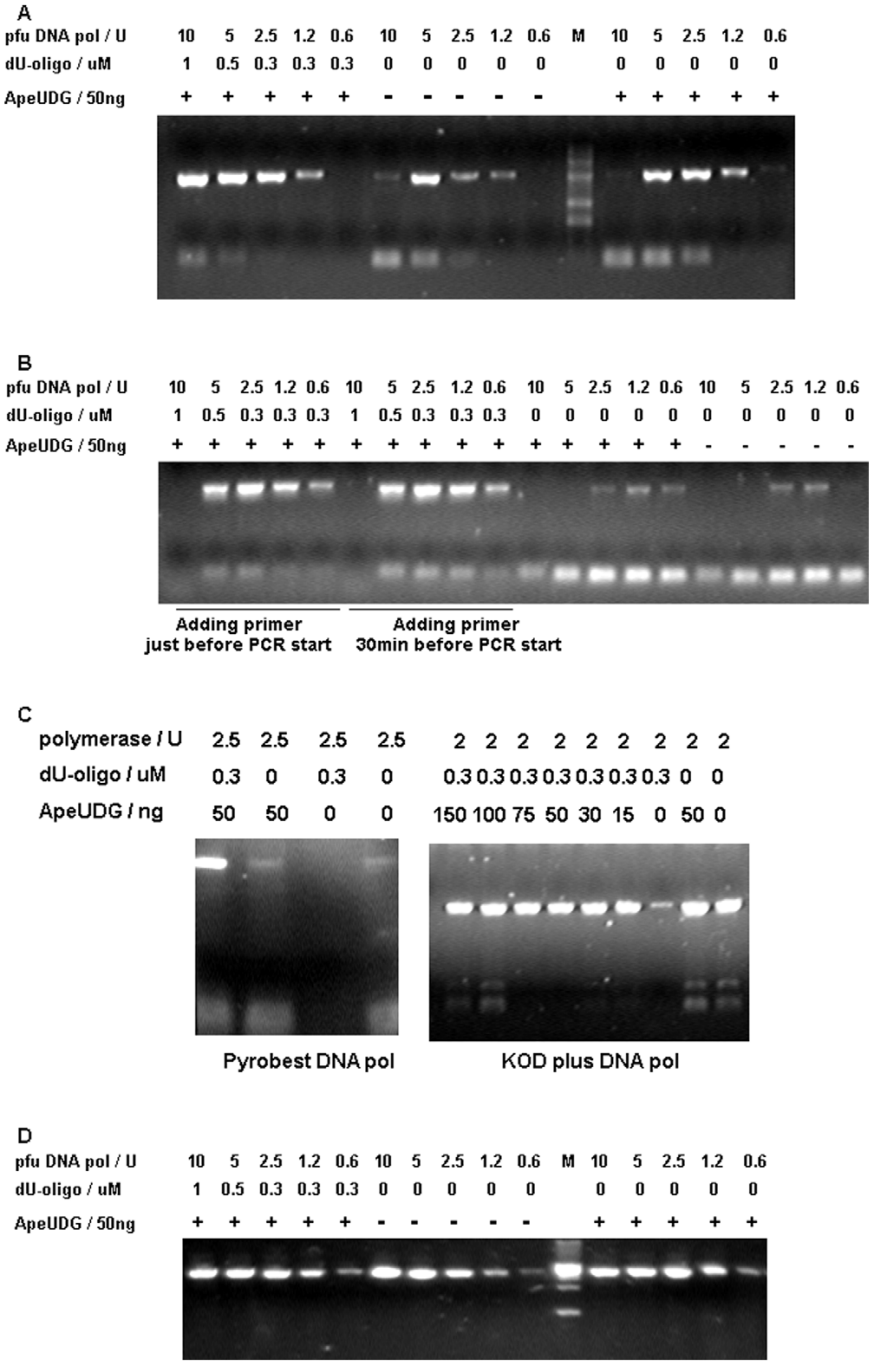
Hot-start PCR of family B DNA polymerases. The PCR of (A, B, and C) ape_0119 and (D) linear pDEST17 fragment was performed in the presence of ApeEndoIV (50 ng). The Pfu DNA polymerase in panel A was from MBI, while that in panel B was a low-quality enzyme from another company. Two other family B DNA polymerases, *Pyrobest* DNA polymerase and KOD DNA polymerase, were used in panel C. Pfu DNA polymerase was used to amplify a 4.6-kb long linear fragments using the pDEST17 vector as template (D). Amplified products were resolved in 1% agarose gel, along with a molecular weight standard DNA ladder (M).

We selected two DNA fragments to test our hot-start PCR strategy. One fragment is ORF ape_0119 (about 1.0 kb) from *A. pernix*, and the other is linear pDEST 17 vector (4.6 kb). The addition of dU-carrying oligo (dU-oligo) and ApeUDG can improve the PCR of ape_0119 (Figs. 7A–7C). In Figs. 7A and 7B, a single addition of ApeUDG increases the yield of both ape_0119 and primer-dimers. However, the addition of both dU-oligo and ApeUDG not only increases the yield of ape_0119, but also decreases the generation of primer-dimers. Another problem is that the excess Pfu DNA polymerase inhibits PCR, which may result from the strong digestion of primers by 3′ exonuclease activity. Interestingly, the inhibition of PCR by excess Pfu DNA polymerase is alleviated largely in the presence of both dU-oligo and ApeUDG. dU-oligo and ApeUDG improve PCR by low-quality Pfu DNA polymerase much better (Fig. 7B) than PCR by good common commercial Pfu DNA polymerase (Fig. 7A), indicating dU-oligo and ApeUDG are much better enhancer for the performance of low quality commercial Pfu polymerase.

The addition of dU-oligo and ApeUDG can also improve the PCR of ape_0119 by two other family B DNA polymerases: *pyrobest* DNA polymerase and KOD DNA polymerase (Fig. 7C). The extent of improvement of *pyrobest* DNA polymerase is much better than that of KOD DNA polymerase. If more ApeUDG is added, some primer-dimers are generated during PCR by KOD DNA polymerase. This is because excess ApeUDG can activate some polymerase by removing dU from dU-oligo before PCR starts, so it is better to ensure the proper addition of ApeUDG so that only the optimum amount will be present in the PCR mixture.

The addition of dU-oligo and ApeUDG improves the PCR of long-fragment pDEST17 vector to a lesser extent. Considering that the PCR of pDEST17 by Pfu DNA polymerase alone is very good (high yield and free of primer-dimers), improvements by dU-oligo and ApeUDG are less, except for the increase of PCR yield under conditions with less polymerase.

### Hot-start PCR of some *A. pernix* genes

**Figure 8:**
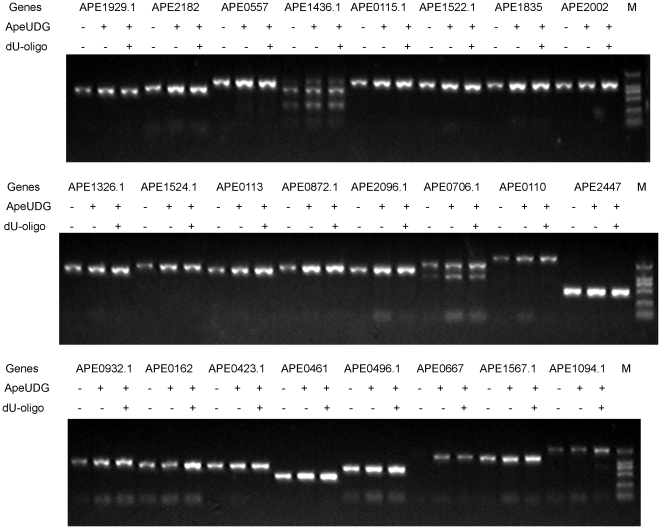
Hot-start PCR of 24 genes from *A. pernix* by Pfu DNA polymerase. Hot-start amplification of 24 genes from *A. pernix* was performed using our method in the presence of 50 ng ApeEndoIV and 5 ng *A. pernix* genomic DNA. The PCR was carried out as follows: predenaturation of 5 min at 95°C and 30 cycles at 94°C for 30 s, 55°C for 30 s, and 72°C for 4 min, followed by a 5-min extension at 72°C. 2.5 U Pfu DNA polymerase, 50 ng ApeUDG, and 0.3 M dU-oligo were used. Amplified products were resolved in 1% agarose gel, along with a molecular weight standard DNA ladder (M).

We amplified 24 DNA fragments to further confirm the validity of our hot-start PCR method (Fig. 8). In the PCR amplification of 24 targets, most fragments are highly amplified and significantly less amounts of primer-dimers are generated. Of course, the addition of ApeUDG and dU-oligo has no distinct improvement on the amplification of several fragments (ape1436.1, ape0706.1, ape0932.1, ape0162, and ape0496.1). For the amplification of ape1436.1 and ape0706.1, the yields of target and unwanted fragments are both increased. The generation of undesired DNA fragments may be the result of the primer not being specifically annealed with the template during the thermo-cycle. Therefore the optimization of annealing temperature is required to delete the unwanted band for the amplification of ape1436.1 and ape0706.1. More primer-dimers are generated from the amplification of ape0932.1, ape0162, and ape0496.1. This may be because the primer-dimers still stably pair at the start of PCR.

## Discussion

At optimal growth temperatures of hyperthermophilus, spontaneous deamination of cytosine/adenine occurs at several orders of magnitude higher than at more moderate temperatures [1]. Hyperthermophilic microbes have developed a highly efficient repair pathway to counter cytosine/adenine deamination. These proteins include various UDGs and dUTPases responsible for dU repair [2]–[6], endonuclease V and Alka responsible for the repair of hypoxanthine (the deamination products of adenine) [30]–[32], and Pfu DNA polymerase [7]–[9], [27], [28], [33]. However, it is not clear how these proteins cooperate and interact to complete this repair.

Previous reports have shown that Pfu DNA polymerase binds tightly to the dU/dI-carrying template and stops the extension by four nucleotides before the dU/dI. It is speculated that this activity prevents the misincorporation of adenine opposite uracil or cytosine opposite hypoxanthine, which results in the transition mutation of G:C to A:T or vice versa. However, most dU/dI should be repaired prior to DNA replication. What is the real *in vivo* function of tightly binding dU/dI by Pfu DNA polymerase? Maybe it functions as a read-ahead recognition and detection of dU/dI and recruits other proteins to fulfill the repair. Recent studies have shown that the family IV UDG and AP endonuclease interact with PCNA, and this activity is promoted by PCNA [34], [35]. The fact that Pfu DNA polymerase is responsible for DNA synthesis in the cell and interacts with PCNA means that PCNA, UDG, AP endonuclease IV, and Pfu DNA polymerase may cooperate to fulfill the repair of dU damage.

PCR is a very important technology in molecular biology. Many thermostable DNA polymerases are used to amplify DNA, and their enzymic characteristics decide whether or not a PCR-reaction with special amplification performance can be fulfilled. Since the discovery of PCR, many efforts have been made to enhance various aspects of its performance, such as the polymerases' processivity, the yield and specificity of amplified product. Some DNA-binding proteins have been used to improve polymerases' processivity and efficiently amplify long DNA fragments. These processivity factors include PCNA, RFC35, RFC53, and Sso7d [36], [37]. In some cases, the processivity factors or their motifs, such as helix–hairpin–helix (HhH), are covalently linked to DNA polymerase [38], [39]. These modifications on polymerases' processivity are useful for most DNA polymerases. However, for family B DNA polymerase from archaea, the modification cannot dissolve the inhibition of dU on polymerases, which discounts its performance in PCR. dUTPase is used to dissolve the inhibition of dU by the hydrolysis of dUTP generated in PCR [2]. However, dUTPase cannot delete the inhibition completely, since dU is also produced from the deamination of dC in DNA.

In the current study, we shows that thermostable UDG and endoIV can delete the inhibition of dU, which results from both deamination of dC and incorporation of dUMP, on family B DNA polymerase to finally correct the uracil error generated during PCR. Considering that most polymerases used in PCR are high-fidelity family B DNA polymerases, the addition of thermostable UDG and endoIV has broad applications in PCR. Of course, the best PCR results are obtained when UDG and endoIV are combined with dUTPase, as previously report [2].

Although thermostable UDG can improve PCR yield via the deletion of the inhibition of dU on family B DNA polymerase, it also increases the yield of undesired DNA fragments. In the current study, we presented a new hot-start PCR strategy to prevent undesired DNA synthesis by blocking the polymerase activity with a dU-carrying oligonucleotide during PCR set-up. PCR is initiated by the addition of thermostable UDG and heating. Our hot-start PCR has several advantages. First, the blocking of polymerase activity is very stable if the thermostable UDG is not added into the PCR mixtures. Thus, it is very convenient to store the blocked family B DNA polymerase before initiating PCR. Second, the dU-carrying oligonucleotide is a common blocker for all family B DNA polymerases, unlike the polymerase-specific antibody [16]. Third, the dU-carrying oligonucleotide is cheaper and easier to prepare than the antibody to the polymerase [16]. Fourth, the thermostable UDG functions as not only an enhancer of DNA synthesis to increase PCR yield, but also an activator of DNA polymerase. Fifth, the combination of UDG and endoIV can correct the error on the uracil base and generate the best-amplified product by improving the fidelity of DNA synthesis during PCR.

During PCR the uracil is produced from two reactions: one is the spontaneous deamination of dC in DNA, and the other is the incorporation of dUMP, which is derived from the deamination of dCTP, into the new synthesized DNA strand by DNA polymerase. The uracil resulting from incorporation of dUMP has no harmful effect on PCR by family A DNA polymerase, such as Taq DNA polymerase. However, the uracil derived from deamination of dC base in DNA will resulted in G:C to A:T transition if not repaired before next round DNA amplification. The use of repair proteins of UDG and endoIV from *A. pernix* in PCR can correct the uracil error generated by the deamination of dC during PCR and hence improve the fidelity of amplified DNA by family A DNA polymerase. Furthermore, 3′ exonuclease of endoIV can supply the deficient proofreading activity of Taq DNA polymerase and improve the fidelity of DNA synthesis. Therefore, improvement of the fidelity of PCR catalyzed by family A DNA polymerase via the combined function of ApeUDG and AP endonuclease is a suitable approach.
